# An Attachment-Based Family Therapy for Anxiety and Depression in Children: A Mixed-Methods Evaluation of BEST-Foundations †

**DOI:** 10.3390/children11121552

**Published:** 2024-12-20

**Authors:** Kim Lee Kho, Andrew J. Lewis, Renita A. Almeida

**Affiliations:** 1 School of Psychology, Murdoch University, Perth, WA 6150, Australia; kim.kho@luminouspsychology.com.au (K.L.K.); renita.almeida@murdoch.edu.au (R.A.A.); 2 Institute of Health and Wellbeing, Federation University Australia, Berwick, Melbourne, VIC 3805, Australia

**Keywords:** attachment, attachment-based family intervention, family therapy, attachment-based intervention, internalising symptoms, childhood depressive symptoms, childhood anxiety

## Abstract

Background/Objectives: Parent–child attachment and family relationships have been identified as risk factors for childhood internalising symptoms such as anxiety and depressive symptoms. This mixed-methods evaluation examined the feasibility of a recently developed attachment-based family intervention, Behaviour Exchange Systems Therapy-Foundations (BEST-F), delivering 16 h of therapy over 8 weeks to treat internalising symptoms in children aged between 3 and 11 years. Methods: The quantitative outcomes of this uncontrolled study of 17 families were based on the parent-reported Child Behaviour Checklist (CBCL) measure, completed at four-timepoints (baseline, pre-, post-intervention, and follow-up), while qualitative data were collected from interviews with participants at follow-up. Results: Pre- and post-BEST-F intervention results demonstrated a significant change in internalising symptoms from the borderline and clinical range to the normal range, with a large effect size (*d* = 0.85). Notably, additional reductions in internalising symptoms were reported two months after cessation of treatment, with a very large effect size (*d* = 1.85). Furthermore, there were substantial reductions in child externalising symptoms and parental mental health symptoms, with large effect sizes ranging from *d* = 0.80 to 1.12. Qualitative reports were consistent with these quantitative findings. Conclusions: These pilot results suggest that children presenting with clinical-range internalising symptoms may benefit from family-based approaches where the parent–child relationship is a focus.

## 1. Introduction

In Australia, approximately 49% of children aged 4 to 11 years old with a mental disorder sought treatment for emotional disorders. Service use was highest among children who presented with a major depressive disorder (MDD) (73%), followed by anxiety disorders (54%) [1]. A recent meta-analysis suggested that worldwide, clinically significant depression and anxiety symptoms in children and adolescents have continued to increase by more than twofold beyond the prevalence rates reported before the COVID-19 pandemic [2]. This supports the need for effective interventions targeting childhood anxiety and depressive disorders.

There is relatively less evidence for interventions of childhood depression and anxiety disorders than for adolescent and adult populations [3,4]. Few treatment options for children experiencing depression and anxiety focus on the broader familial network, despite the well-documented influence of interactions between genetic variability and caregiving/family environmental factors on the transmission of depression and anxiety [5,6,7]. Various family factors, including attachment security, conflict within the family, parental mental health, and family functioning have been reported to share a bidirectional relationship with the risk of children developing depression and anxiety disorders [8,9,10,11,12,13]. A number of intervention studies showed that those interventions which include family members outperformed individual child-only interventions, with a reported increase of 70% in child engagement in treatment [14,15,16].

Attachment-based interventions aim to promote security and organisation in the child’s attachment to their caregivers, thus improving the child’s regulation of emotion potentially reducing vulnerability to childhood depression and anxiety [17,18]. Parental reflective functioning (PRF) is a key concept in such intervention models and refers to a parent’s ability to reflect upon the mental states underlying a child’s actions and emotions, thereby influencing the nature of parent–child interactions and ideally contributing to the child’s ability to self-regulate emotions [19]. Additionally, PRF is thought to be important in promoting secure attachment and facilitating the child’s developing capacity to understand their own mental states and those of others [20].

BEST-Foundations (BEST-F) was developed approximately a decade ago based on the original Behaviour Exchange Systems Therapy (BEST) program, which has been in use in Australia since the 1990s. This manualised intervention was designed to target modifiable family factors to treat clinical or subclinical symptoms of internalising disorders, including depression and anxiety disorders, in children aged between 3 and 11 years [21]. Similar to its predecessors focused on adolescents [22,23,24], this program combines family systems and attachment theory to assist in the early treatment of symptoms of depression and anxiety disorders by building cohesive and strong family connections, improving communication within the family, and increasing parents’ understanding of the child’s mental state.

The first stage of the BEST treatment focuses on strengthening the parent–child relationship by encouraging PRF [19] through several means, including the parental reflective interview (PRI) [25] and video-feedback [26], with the guidance of therapists specifically trained in this model. It also encourages the primary caregiver and child to spend more one-on-one time together through a “Special Time” activity, that is, prescribed periods of relatively unstructured play time where the primary focus is on parent–child interaction and the enjoyment of play. The second stage of treatment shifts to include all family members and is aimed at fostering family connectedness by promoting family cohesiveness and reducing conflict. In this stage, several metaphorical themes are introduced to encourage parental self-care, the modelling of adaptive coping strategies, open communication, and the processing of significant life events experienced as a family to facilitate the promotion of a “secure family base” using a technique called “Bumps in the Road” [27]. The third stage of treatment is future-focused, where the aim is to create a sense of purpose and direction in family life through family rituals [28]. Each of these components of the BEST program and the BEST program logic have been presented in detail in previous publications [22,29,30,31,32,33,34,35].

The first BEST-F pilot trial was conducted at Deakin University, Victoria, where preliminary results showed positive improvements in pre–post child internalising symptoms, and these improvements were sustained at follow-up [21]. The first BEST Foundations study by Benstead [21] was conducted in a university setting and examined outcomes for eight families and reported large effects for child internalising symptoms pre–post (*d* = 0.83) and maintained at follow-up (*d* = 0.92). This initial evaluation also reported improvements in externalising problems and total problems. The current study is a larger evaluation in a different clinical setting with different therapists. However, this study has similar aims to investigate the efficacy of BEST-F in reducing internalising symptoms of depressive and anxiety disorders in children aged between 3 and 11 years. A notable difference is that the current study recruited its sample from children presenting for mental health services in a community clinic.

In the current study, it was predicted that children who participated in BEST-F would show significant reductions in internalising symptoms as measured by the Child Behavioural Checklist (CBCL) comparing pre- and post-intervention scores, and these reductions would be sustained at follow-up. We predicted that effect sizes would be consistent with the initial pilot trial [21]. Furthermore, changes in child externalising symptoms and other problems, as measured by the CBCL, parental mental health symptoms, and caregiving behaviours, were also evaluated as secondary outcomes. Similarly, we predicted significant reductions in child externalising symptoms and other problems, parental mental health symptoms, and caregiving behaviours when comparing pre- and post-intervention scores, and that these reductions would be sustained at follow-up. In addition, qualitative methods were used to understand the participants’ lived experiences of the treatment and process of therapeutic change [36].

## 2. Materials and Methods

### 2.1. Participants

**Figure 1:**
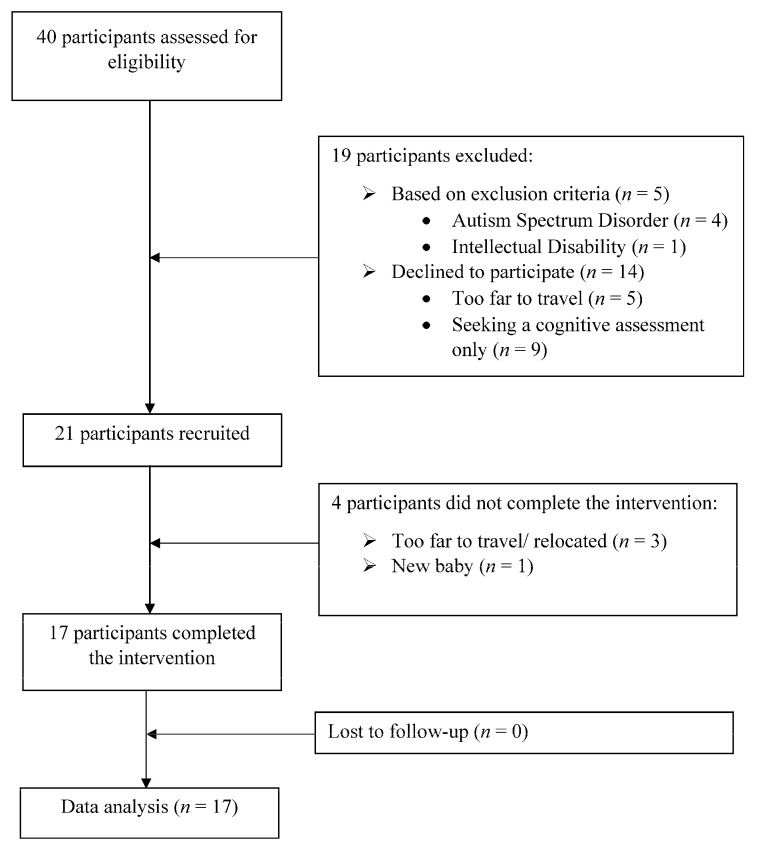
Flow diagram of BEST-Foundations participant recruitment and attrition.

Forty children aged between 3 and 11 years and their families who had self-referred to the Murdoch Psychology Clinic in Perth, Western Australia, and who were on a waitlist for a mental health service, were recruited. Reasons for attrition are outlined in Figure 1. Inclusion criteria included children aged between 3 and 11 years who presented with subclinical or clinical level depressive- and anxiety-related symptoms as screened using the Children Mood and Anxiety Scale (CMS), which comprised the Preschool Feelings Checklist (PFC) [37] and the Revised Preschool Anxiety Scale (PAS) [38], via telephone. Exclusion criteria included children presenting with neurodevelopmental disorders, significant cognitive impairment, or other severe mental health diagnoses requiring inpatient treatment, as well as parents presenting with severe mental illness requiring inpatient treatment or impairing their capacity to participate in the program.

The final sample consisted of 17 children, including 9 girls and 8 boys (age: *M* = 8.88 years; *SD* = 1.69) and their primary caregivers, their mothers (*n* = 17; age: *M* = 40.18 years; *SD* = 7.26). All participants attended the eight weekly sessions and completed the full BEST-F program. Table 1 presents the demographic characteristics of the participants.

### 2.2. Design

A mixed-methods design was utilised, where both qualitative and quantitative data were given equal weighting [39]. In the evaluation of BEST-F, the quantitative and qualitative data were collected and analysed independently, but the overall findings were integrated to cross-validate findings and general conclusions [40]. Specifically, the efficacy of BEST-F was examined using a multiple baseline design, with quantitative data collected at four-timepoints: (i) initial entry to the study (baseline); (ii) commencement of treatment which occurred four weeks after baseline (pre-); (iii) conclusion of treatment (post-); and (iv) at 8-weeks follow-up. This design allowed for the participants to act as their own control by comparing a “no-treatment” period between baseline and commencement to changes which occurred from commencement to post-treatment and follow-up.

### 2.3. Measures

Child Behavioural Checklist for Ages 6–18 (CBCL): The CBCL [41] is a 113-item parent proxy report that assesses child emotional and behavioural difficulties. The items are rated on a 3-point Likert scale that generates eight syndrome scales and three composite scales, including internalising problems, externalising problems, and a total problems aggregate scale. (Achenbach and Rescorla, 2001 [41]). Standard CBCL *T*-scores above 65 are either within the borderline (65 ≤ *T* ≤ 69) or the clinical range (*T* ≥ 70) [41]. The CBCL has demonstrated excellent internal consistency, α = 0.89 for the internalising scale, α = 0.93 for externalising scale, and α = 0.96 for total problems scale [42].

State-Trait Anxiety Inventory (STAI): The STAI [43] was administered to parents. It is a self-report questionnaire that measures the presence and severity of caregiver anxiety and distress. The two 20-item subscales examine generalised tendency to experience anxiety (T-Anxiety) and current state of perceived anxiety (S-Anxiety) that were rated on a 4-point frequency scale. Higher scores indicate greater anxiety, with a cut-off point of 39–40, which suggests a detectable clinical significance in symptoms [44]. The STAI has demonstrated high internal consistency, α = 0.86 to 0.95, and test–retest reliability, *r* = 0.65 to 0.75, over a 2-month interval [45]. This measure has also demonstrated discriminant, construct, and content validity [45,46,47].

Edinburgh Depression Scale (EDS): The Edinburgh Postnatal Depression Scale [48] was administered to parents. It is a 10-item self-report measure to examine symptoms of emotional distress during pregnancy and in the postnatal period. In non-post-natal populations, the scale is referred to as the Edinburg Depression Scale (EDS), as is the case in this study. The EDS was used to assess caregivers’ current emotional distress levels on a 4-point Likert scale [48]. The EDS has demonstrated high internal consistency coefficients ranging from 0.73 to 0.92, as well as sensitivity and specificity of 0.87 and 0.91, respectively [48]. Furthermore, the EDS has been validated as a generic measure of depression among non-postnatal women with older children [49] and fathers [50].

Caregiving Helplessness Questionnaire (CHQ): The CHQ [51] was administered to parents. It is a 26-item questionnaire designed to measure disorganised caregiving in mothers of children aged between 3 and 11 years. This measure consists of three subscales: (1) caregiving helplessness; (2) caregiver–child frightened; and (3) child caregiving. All responses were recorded on a 5-point Likert scale, where a higher score reflects more disorganised caregiving patterns. The CHQ demonstrated good factor structure and internal consistency ranging from α = 0.64 to 0.85, as well as convergent and discriminant validity [51,52].

Qualitative Survey Questions: A set of 14 questions, included as a purpose-designed evaluation measure within the BEST-F manual, were used in the follow-up session as prompts for recorded discussions with parent and child participants [21].

### 2.4. Procedure

Upon receiving ethics approval from Murdoch University Human Research Ethics Committee, all potential participants were reviewed for eligibility from the waitlist of the Murdoch Psychology Clinic and screened for eligibility using the CMS. Following this, written informed consent was obtained at the initial intake session. Each individual family attended eight weekly 2 h sessions (the first session being the initial intake session), and a follow-up session 8-weeks post-intervention (recorded semi-structured interview). This intervention required the primary caregiver and child to attend all sessions, with the rest of the family including a second parent and siblings participating from session four onwards. The therapists were all provisionally registered psychologists undergoing postgraduate training in clinical psychology. All therapists were required to attend the BEST-F training program over a two-day period, to observe or co-facilitate at least one BEST intervention, and to attend fortnightly group supervisions by the program developer/experienced supervisor where video-recordings of the sessions were reviewed. All video recordings, de-identified client information, and research data were stored in a locked cabinet.

### 2.5. Data Analysis

Quantitative data were analysed using IBM SPSS Statistics (Version 27). To test the hypotheses on outcomes of children and parent mental health outcomes, as well as caregiving behaviours, within-subject repeated-measures ANOVA analyses were utilised to investigate significant differences between the timepoints at *p* < 0.05. In cases where results of repeated-measures ANOVA were significant, post hoc pairwise comparisons were examined to determine significant differences across the timepoints. In addition, effect sizes using Cohen’s *d* were calculated [53].

Qualitative data were analysed based on the phenomenological approach of the participants’ subjective experiences of the intervention. Specifically, qualitative thematic analysis based on descriptive phenomenology was utilised to gain insight into the participants’ experience of the program [54]. The audio-recorded interview was transcribed verbatim then coded and organised into meaningful and coherent themes that were relevant to the participant’s experiences of the intervention.

Statistical power analysis was conducted for sample size estimation based on the statistics from this study. The “G*Power” program [55] was utilised to calculate the sample size needed to detect some level of effect. The effect size of this study was based on the prior study by Benstead and set at Cohen’s *d* = 0.85 for pre- and post-child internalising symptoms. Using α = 0.05 and 80% power analysis indicated that the projected sample size was approximately *n* = 6 for a within-subjects comparison. As such, the present study’s sample size of *n* = 17 was adequately powered.

## 3. Results

### 3.1. Primary Outcome: Child Internalising Symptoms

In order to examine changes in child internalising symptoms, repeated-measures ANOVA using parent-reported CBCL composite- and sub-scales across the four-timepoints and effect size calculation were conducted, and the results are presented in Table 2.

Children’s internalising CBCL *T*-scores reduced from pre- to post-intervention, and further reduction was observed from post-intervention to follow-up. Results of repeated-measures ANOVA indicated that there was a significant reduction in CBCL total score from baseline (*M* = 67.24l; *SD* = 8.20), which was within the borderline clinical range, to normal range at follow-up (*M* = 54.29; *SD* = 7.95); i.e., *F*(3,14) = 15.21, *p* < 0.001, η_p_^2^ = 0.77. Specifically, the results revealed a significant reduction in child internalising CBCL scores from baseline (*M* = 68.29; *SD* = 10.46), which was within the borderline clinical range, to normal range at follow-up (*M* = 54.94; *SD* = 7.33); i.e., *F*(3,14) = 15.78, *p* < 0.001, η_p_^2^ = 0.77. Follow-up pairwise comparisons revealed significant reductions in child internalising symptoms from baseline to post-intervention (*MD* = 4.59, *p* = 0.002), baseline to follow-up (*MD* = 13.35, *p* < 0.001), and pre-intervention to follow-up (*MD* = 12.94, *p* = < 0.001). There was no significant improvement in CBCL internalising scores between baseline and pre-intervention (*MD* = 0.41, *p* = 1.00), indicating the stability of the child internalising scores during the control period where there was no intervention. In addition, the calculation of intervention effect size revealed large effect sizes between pre- and post-intervention (*d* = 0.85) and between pre-intervention and follow-up (*d* = 1.85).

### 3.2. Secondary Outcomes

Other Child Mental Health Symptoms: A significant reduction in parent-reported child externalising CBCL scores was found from baseline (*M* = 62.76; *SD* = 9.57) to follow-up (*M* = 52.59; *SD* = 9.37); i.e., *F*(3,14) = 9.71, *p* < 0.001, η_p_^2^ = 0.68. Pairwise comparisons indicated significant reductions in child externalising symptoms from baseline to post-intervention (*MD* = 7.59, *p* = 0.001), baseline to follow-up (*MD* = 10.18, *p* < 0.001), and pre-intervention to follow-up (*MD* = 9.35, *p* = 0.001). There was no significant change in CBCL externalising scores between baseline and pre-intervention (*MD* = 0.82, *p* = 0.99) indicating stability of child externalising scores during the control period. Furthermore, calculation of intervention effect size demonstrated a moderate effect size between pre- and post- intervention (*d* = 0.65) while a large effect size existed between pre-intervention and follow-up (*d* = 0.96). ANOVA analyses of CBCL externalising subscales revealed a significant decrease in scores for aggressive behaviour, i.e., *F*(3,14) = 12.97, *p* < 0.001, η_p_^2^ = 0.74, but not rule-breaking behaviour, i.e., *F*(3,14) = 3.07, *p* = 0.06, η_p_^2^ = 0.40. In addition, analyses of other CBCL subscales found significant decreases in baseline to follow-up scores for subscales including social, thought, and “other problems”, but not attention problems, i.e., *F*(3,14) = 1.99, *p* = 0.16, η_p_^2^ = 0.30.

Parent Mental Health Outcomes: Table 3 displays means and standard deviations of EDS and STAI and the results of repeated-measures ANOVA to examine changes in parental depressive and anxiety symptoms and the effect size calculation across the four-timepoints. Results of ANOVA analyses found a significant reduction in parent’s EDS scores from baseline (*M* = 13.53; *SD* = 5.27), which was within the clinical range, to follow-up (*M* = 8.24; *SD* = 3.70), which was within the normal range (*F*(3,14) = 12.37, *p* < 0.001, η_p_^2^ = 0.73). Pairwise comparisons revealed significant reductions in parent depressive symptoms from baseline to post-intervention (*MD* = 5.18, *p* < 0.001), baseline to follow-up (*MD* = 5.29, *p* = 0.003), pre- and post-intervention (*MD* = 4.82, *p* < 0.001), and pre-intervention to follow-up (*MD* = 4.94, *p* = 0.004), as well as insignificant changes in EDS scores between baseline and pre-intervention (*MD* = 0.35, *p* = 1.00), indicating the stability of the EDS scores during the control period. Moreover, large effect sizes were demonstrated between pre- and post-intervention (*d* = 1.13) and between pre-intervention and follow-up (*d* = 1.12). As for parents’ STAI-S scores, ANOVA analyses indicated a significant reduction in STAI-S scores from baseline (*M* = 43.41; *SD* = 7.75), which was within the clinical range, to follow-up (*M* = 38.18; *SD* = 5.77), which was within the normal range; i.e., *F*(3,14) = 3.78, *p* = 0.04, η_p_^2^ = 0.45. Pairwise comparisons found significant reductions in parents’ state-anxiety symptoms from pre-intervention to follow-up (*MD* = 6.06, *p* = 0.02) and insignificant changes in STAI-S scores between baseline and pre-intervention (*MD* = 0.83, *p* = 1.00), indicating the stability of the STAI-S scores during the control period. Moreover, calculation of intervention effect size found a medium effect size existed between pre- and post-intervention (*d* = 0.75), while a large effect size existed between pre-intervention and follow-up (*d* = 1.06). Similarly, parents’ STAI-T scores reduced significantly from baseline (*M* = 42.12; *SD* = 5.77) to follow-up (*M* = 38.53; *SD* = 4.68); i.e., *F*(3,14) = 8.30, *p* < 0.001, η_p_^2^ = 0.64. Pairwise comparisons revealed a significant reduction in parents’ trait-anxiety symptoms from pre-intervention to follow-up (*MD* = 3.82, *p* = 0.003) and insignificant changes in STAI-T scores between baseline and pre-intervention (*MD* = 0.24, *p* = 1.00), indicating stability of STAI-T scores during the control period. A small effect size existed between pre- and post-intervention (*d* = 0.15), while a moderate to large effect size existed between pre-intervention and follow-up (*d* = 0.70).

Parent Caregiving Behaviours: Table 4 displays means and standard deviations of the CHQ total and subscale scores, the results of repeated-measures ANOVA in examining changes in caregiving behaviours, and the effect size calculation across the four-timepoints. ANOVA analyses indicated that there was a decrease in CHQ total scores from baseline (*M* = 45.41; *SD* = 8.16) to follow-up (*M* = 38.00; *SD* = 8.31); i.e., *F*(3,14) = 3.46, *p* = 0.04, η_p_^2^ = 0.43. Pairwise comparisons found a significant reduction in CHQ total scores from pre-intervention to follow-up (*MD* = 6.71, *p* = 0.30) and insignificant changes in CHQ total scores between baseline and pre-intervention (*MD* = 0.71, *p* = 1.00), indicating stability of CHQ scores during the control period. In addition, the calculation of the intervention effect size found that a minute effect size existed between pre- and post-intervention (*d* = 0.05), but a large effect size existed between pre-intervention and follow-up (*d* = 0.80). Nonetheless, analyses of CHQ subscales showed that the significant decrease in scores from baseline (*M* = 11.29; *SD* = 3.33) to follow-up (*M* = 8.71; *SD* = 1.96) only existed in the caregiver–child frightened subscale; i.e., *F*(3,14) = 3.19, *p* = 0.04, η_p_^2^ = 0.41. Pairwise comparisons found a significant reduction in caregiver–child frightened subscale scores from baseline to follow-up (*MD* = 2.59, *p* = 0.04), with no significant reduction between baseline and pre-intervention (*MD* = 1.41, *p* = 0.42), indicating stability of the subscale scores during the control period where there was no intervention.

### 3.3. Qualitative Outcomes

Thematic analysis of the interview conducted at the follow-up timepoint was conducted. Four core themes emerged, focused on participant perceptions of change and the participants’ experiences of BEST-F. These were Special Time, video-feedback, parental reflective interview (PRI), and a supportive therapeutic climate.

Special Time: All participants considered “Special Time” as the most helpful technique and indicated they will continue implementing at home. Parent participants reflected that implementing Special Time had improved the parent–child bond and communication as it allowed them to spend quality time together, build a foundation of partnership, and give the child space to express feelings. Child participants reported that the structure allowed them to spend undisturbed time with their parent.

*Parent participant: “I have a different role during Special Time as he [child] leads the play and the way we interact has changed positively.”*

*Child participant: “I look forward to this daily…playing with mum, just both of us.*

Video-Feedback: The majority of parent participants described video-feedback as confronting but effective in helping them become more aware of their child’s cues. They described other benefits gained, including increased awareness of their responses and those of their children, and increased confidence in modelling positive communication.

*Parent participant: “Now I’m more aware of how I respond to him and it has made incredible differences in how he reacts to me.”*

Parental Reflective Interview (PRI): Parent participants considered the PRI as the most challenging but helpful technique as this experience reportedly allowed them to become more aware of, and to understand, their behaviours and reactions as a consequence of their internal experiences.

*Parent participant: “It was difficult when we talked about my childhood. Now, I can see why that was important and it helped me make sense of my relationship with him [child].”*

Supportive Therapeutic Climate: Participants emphasised the importance of the therapist’s ability to build and maintain good rapport with both parent and child. Such rapport contributed to building and maintaining a supportive therapeutic environment. The majority of parent participants described that a crucial factor contributing to their feeling of safety and trust is the therapist’s ability to express empathy, care, and understanding of their situation. Furthermore, child participants characterised their relationship with the therapist as “safe” and collaborative, which helped them build confidence in communicating with their parents. In addition, participants highlighted the importance of facilitating difficult conversations between family members. All participants suggested that the therapists were able to “contain” difficult conversations by accurately identifying and reflecting feelings and content of the conversation, as well as setting appropriate boundaries.

*Parent participant: “I felt heard and seen by you [therapist] when we talked about what I’ve experienced in the past. I felt safe and that changed everything for me, and my family could tell the difference too.”*

*Child participant: “I feel it’s safe talking about it here…now I can tell mum when I am upset if something happens at school or with dad.”*

## 4. Discussion

The primary aim of this study was to examine the efficacy of a novel attachment-based family intervention, BEST-F, using mixed-methods evaluation. As predicted, the results of this study revealed that children who participated in BEST-F demonstrated a significant reduction in internalising symptoms at post-intervention, and these symptoms continued to reduce at follow-up. In addition, this study evaluated changes in child externalising symptoms and other problems, parental mental health outcomes (depression and anxiety), and improved caregiving behaviours. In line with the hypotheses, the results of this study also revealed that children who participated in BEST-F showed significant reductions in externalising symptoms and total problems at post-intervention, and these symptoms further reduced at follow-up. As predicted, the same was found for parents who participated in BEST-F, demonstrating significant reductions in symptoms of anxiety and depression, as well as disorganised caregiving behaviours at post-intervention, with further reductions at follow-up.

The findings of this study revealed a significant reduction in symptoms of internalising disorders, including depression and anxiety, at post-intervention, and that these symptoms continued to reduce at 8-weeks follow-up. More importantly, the significant clinical changes moved children from a borderline clinical range during the control period to a normal range at post-intervention. The intervention produced significant reductions in child internalising and externalising symptoms, as well as total problems during the intervention period, with large effect sizes observed across the main CBCL composite scales (*d* = 0.81 to 1.85). These results were better than those of the initial pilot study, which found significant improvements in child internalising and externalising symptoms, as well as total problems, with effect sizes ranging from small to medium across the main CBCL composite scales (*d* = 0.30 to 0.60) [21]. Generally, the results of the current study show similar patterns to the previous and smaller study conducted by Benstead. Combined, these two studies show that the treatment effects of BEST-F can be sustained in different clinical settings and can be undertaken by a range of well-trained and qualified therapists.

More importantly, the additional reductions observed at the 8-weeks follow-up signifies that treatment effect increased after treatment has concluded, thus suggesting that families were able to continue implementing important systemic changes even after treatment has ceased [23]. Therapeutic changes likely continued beyond the active therapy period because families continued to implement changes in their patterns of communication and emotional regulation [56,57]. These results were expected given the strong focus of BEST-F in promoting an improved family environment by improving communication, decreasing conflict, and improving family functioning through the use of attachment and systems models. These findings support the therapeutic strategy that targeting these specific family factors throughout the course of a family-based treatment can produce improvements in child mental health symptoms.

BEST-F acknowledges that parental mental health is strongly intertwined with other family factors and has a bidirectional relationship with childhood depression and anxiety, hence the importance of a whole-of-family approach. Parent outcomes, including symptoms of parental depression (*d* = 1.12) and anxiety disorders (*d* = 0.70 to 1.06), significantly reduced from a clinical range to a normal range at post-intervention, with further symptom reduction at follow-up. These findings were similarly found in previous BEST trials, suggesting that the intervention model facilitates improvements in parental mental health symptoms including stress, depression, and anxiety [23,30,58]. These results differed from the results of the initial BEST-F pilot study, which revealed no significant improvements in parental mental health [21], possibly reflecting a higher degree of clinical severity in that sample which was clinic-referred. According to family systems theory, changes in any part of the family system, whether in the parent, child, sibling, or communication patterns, will impact other parts of the family system [59]. Therefore, by targeting relationships within the family rather than the individual child or parent, positive changes in the family’s functioning and environment are expected [56].

In addition, the results of this study further demonstrated promise in the reduction of disorganised caregiving behaviours at post-intervention, with additional reduction at follow-up and the large effect size observed (*d* = 0.80). Again, these results were an improvement on the results of the initial pilot study that found no significant improvements in caregiving behaviours [21]. Upon closer investigation, parent participants reported feeling less frightened of, or frightening to, their child after the intervention. George and Solomon [60] posited that helpless caregivers often experience feelings of fear and reduced competence in their ability to provide security to their children.

Overall, the qualitative feedback received from participants was largely positive, and the results of qualitative analysis supported the feasibility of BEST-F as a promising and acceptable treatment model for families with children aged between 3 and 11 years presenting with internalising symptoms of depression and anxiety disorders. Qualitative reports suggested that participants derived considerable benefits at both dyadic and systemic levels. These results further revealed major BEST-F elements that participants found helpful in this change process, including “Special Time”, video-feedback, PRI, and a supportive therapeutic climate. A synthesis of the quantitative and qualitative results suggested that children within the subclinical and clinical range of disorders, as measured by the CBCL, and their family responded well to this treatment.

Our findings around the specific components of effective treatment may be of special interest to clinicians. Parent participants shared that video-feedback was useful in helping them become more aware of their child’s cues and responding more sensitively to their children. Video-feedback has emerged as a powerful tool in attachment-based interventions and has consistently proven effective in promoting positive changes in the dyadic relationship in a short time frame [61,62,63]. During the video-feedback session in BEST-F, the therapist carefully selected and then replayed selected sections of a “play” video to highlight specific parent behaviours and the child’s consequent reactions for reflective discussion [28]. Through video-feedback, parents observed themselves “from the outside” and obtained a different perspective on their dyadic relationship by watching specific parts of the video [64]. Furthermore, the use of video-feedback helped foster PRF in the presence of a trained therapist who actively assisted parents with the development of this capacity in relation to specific interactions with their child [65,66]. Past meta-analyses have supported the use of such techniques in increasing parenting sensitivity (*d* = 0.33 to 0.37); improving positive parenting behaviours (*d* = 0.47); promoting secure attachment (*d* = 0.20); and reducing internalising difficulties (*d* = 0.33) [67,68].

Another technique used in BEST-F that aimed to improve parental reflective function was the parental reflective interview (PRI), a carefully structured interview designed to orient the parent participant to attachment themes and their own attachment histories, which was conducted in session one. Despite expressing challenges in completing the PRI due to the resurfacing of emotions connected to their own experiences of being parented, parents reported perceived improvements in their reflective functioning (RF), as described by increased awareness and understanding of their own and their child’s behaviours and reactions. This is consistent with the results of a recent single-blind RCT that evaluated the PRI specifically and found that participants who were administered the PRI showed a significant increase in general RF with large effect size (*d* = 0.77), while the control group that was administered the Mini International Neuropsychiatric Interview for Children and Adolescents—Parents Version (MINI-KID-P), a diagnostic interview, showed a decline in general RF [25]. This positive result provides support for the use of Slade’s [69] proposed therapeutic techniques for developing RF as embedded in the PRI, including the facilitation of “wondering” through therapy and focusing on intentions and internal mental states underlying behaviour, rather than correcting parenting behaviour as such. Consequently, parents can become more attuned to their child’s attachment needs and are better able to assist their child with the development of the child’s RF capacity and emotional regulation [70,71].

At the current stage of the evaluation of BEST-F, this trial provides numerous advantages, including further refinement of the training and evidence base for this approach. [72]. In addition, the use of mixed-methods analyses contributes to the strength of this study. The combination of both quantitative and qualitative outcomes enhances the strength and application of these findings [73]. Moreover, the inclusion of direct feedback from participants who have completed BEST-F allows for the evaluation of real-world effectiveness and provides valuable participant lived experiences [74]. Furthermore, the use of well-validated and reliable outcome measures represents a strength of this study [75].

However, this study was constrained by the nature of small scale and uncontrolled studies. Additionally, the absence of randomisation to an active control group limits this study as non-randomisation does not account for systemic differences between groups, thus possibly affecting the interpretation of these findings [76]. Furthermore, this study incorporated only one follow-up session at 8 weeks post-intervention; this limits the capacity of the current study in evaluating longer-term outcomes of the intervention that may be evident due to continued systemic changes [77]. In this study, the exclusive use of parent-reported measures also limits the understanding of these findings. Multiple informants, including a teacher report, could provide a more helpful insight into child mental health outcomes in several contexts [78]. Nonetheless, the CBCL, in general, is not a diagnostic measure as it only intends to assess problem behaviours in children [79]. Perhaps the incorporation of a structured clinical diagnostic interview could be considered in addition to the CBCL. Therefore, future studies should consider the randomisation of the groups, an active control group, multiple follow-up measurements, multiple informants, and a structured clinical diagnostic interview to allow for a more comprehensive understanding of treatment effects.

This study aimed to evaluate the feasibility of BEST-F, a novel attachment-based family intervention. The findings of this study demonstrated that BEST-F showed promise in reducing symptoms of child psychopathology, parental mental health symptoms, and improved caregiving behaviours. Moreover, these improvements were both maintained and further improved over time, as observed at the 8-weeks follow-up. Taken together with qualitative analysis of participant experiences, BEST-F has the potential to become an effective, feasible, and acceptable model of intervention for families with children aged between 3 and 11 years who present with symptoms of depression and anxiety disorders. The findings of this study add to the limited body of literature on attachment-based family interventions designed to treat childhood depression and anxiety disorders.

## Figures and Tables

**Table 1:** Demographic characteristics and frequencies of BEST-Foundations participants.

Characteristics	*N*	Percentage (%)
Child’s Gender		
Male	8	47.1
Female	9	52.9
Annual Caregiver Income		
Under AUD 20,000	5	29.4
AUD 20,000–AUD 50,000	3	17.6
AUD 50,001–AUD 80,000	4	23.5
AUD 80,001–AUD 110,000	1	5.9
AUD 110,001–AUD 140,000	3	17.6
Above AUD 140,000	1	5.9
Marital Status		
Married	8	47.1
De facto	1	5.9
Single parent	2	11.8
Divorced/Separated	6	35.2
Level of Education		
Up to Year 10	4	23.5
Up to Year 12	3	17.6
Completed TAFE or Diploma	9	52.9
Completed Undergraduate Degree	1	5.9

**Table 2:** Means, standard deviations, repeated measures analyses of variance, and effect size of children’s mental health outcomes (CBCL T-Scores) at four timepoints.

CBCL	Baseline		Pre		Post		Follow-Up		*F*(3,14)	*p*	Partial Eta Squared	Cohen’s *d* (Pre–Post)	Cohen’s *d* (Pre-Follow Up)
	*M*	*SD*	*M*	*SD*	*M*	*SD*	*M*	*SD*					
Composite Scales													
Total Score	67.24	8.20	64.47	9.68	58.29	7.89	54.29	7.95	15.21	<0.001	0.77	0.37	0.81
Internalising	68.29	10.46	67.88	6.29	60.35	10.08	54.94	7.33	15.78	<0.001	0.77	0.85	1.85
Externalising	62.76	9.57	61.94	8.31	55.18	9.97	52.59	9.37	9.71	<0.001	0.68	0.65	0.96
Subscales													
Anxious/Depressed	10.18	5.35	8.00	5.62	6.53	4.70	4.12	3.26	7.99	0.002	0.63	0.13	0.45
Withdrawn/Dep	3.65	3.18	2.65	3.06	2.12	2.52	1.53	1.37	4.63	0.019	0.50	0.15	0.15
Somatic Complaints	5.82	3.88	4.64	3.72	3.06	3.17	2.24	1.92	7.73	0.003	0.62	0.15	0.50
Rule-Breaking	4.47	4.24	3.76	4.05	2.00	2.40	1.47	2.18	3.07	0.062	0.40	0.34	0.50
Aggressive	12.82	6.87	10.06	7.00	7.76	5.41	6.00	4.23	12.97	<0.001	0.74	0.10	0.30
Social Problems	5.24	4.27	5.35	3.39	3.47	3.22	2.41	2.37	4.73	0.018	0.50	0.60	1.03
Thought Problems	6.41	3.90	6.24	2.94	3.82	2.30	3.06	2.49	6.18	0.007	0.57	0.87	1.11
Attention Problems	8.76	4.76	8.35	4.77	6.59	4.53	6.00	4.23	1.99	0.162	0.30	0.29	0.43
Other Problems	6.59	3.76	5.06	2.95	3.59	1.84	3.00	2.09	7.01	0.004	0.60	0.15	0.35

**Table 3:** Means, standard deviations, repeated measures analyses of variance, and effect size of parental mental health outcomes (depression and anxiety) at four timepoints.

	Baseline		Pre		Post		Follow-Up		*F*(3,14)	*p*	Partial Eta Squared	Cohen’s *d* (Pre–Post)	Cohen’s *d* (Pre-Follow Up)
	*M*	*SD*	*M*	*SD*	*M*	*SD*	*M*	*SD*					
EDS	13.53	5.27	13.18	4.67	8.35	3.23	8.24	3.70	12.37	<0.001	0.73	1.13	1.12
STAI-S	43.41	7.75	44.24	6.94	39.42	11.09	38.18	5.77	3.78	0.041	0.45	0.75	1.06
STAI-T	42.12	5.77	42.35	6.67	41.82	1.81	38.53	4.68	8.30	<0.001	0.64	0.15	0.70

**Table 4:** Means, standard deviations, repeated measures analyses of variance, and effect size of disorganised caregiving outcomes (Caregiving Helplessness Questionnaire) at four timepoints.

	Baseline		Pre		Post		Follow-Up		*F*(3,14)	*p*	Partial Eta Squared	Cohen’s *d* (Pre–Post)	Cohen’s *d* (Pre-Follow Up)
	*M*	*SD*	*M*	*SD*	*M*	*SD*	*M*	*SD*					
CHQ (Total)	45.41	8.16	44.71	6.88	43.53	9.13	38.00	8.31	3.46	0.043	0.43	0.05	0.80
Caregiving Helplessness	13.82	5.05	13.76	4.72	12.71	5.65	10.76	4.63	2.22	0.131	0.32	0.19	0.63
Caregiver- Child Frightened	11.29	3.33	9.88	2.96	9.65	2.91	8.71	1.96	3.19	0.044	0.41	0.37	0.07
Child Caregiving	20.53	4.65	20.71	3.85	21.65	2.98	20.18	4.88	1.85	0.185	0.28	0.23	0.16

## Data Availability

Data are available by contacting Kim L. Kho, subject to the data management requirements of the study’s ethics approval.
